# Training and Experience of Peer Reviewers: Is Being a “Good Reviewer” a Persistent Quality?

**DOI:** 10.1371/journal.pmed.0040144

**Published:** 2007-03-27

**Authors:** Ignacio García-Doval

After reading your interesting paper [1], I think that all editors will feel a bit disappointed that there are no magic answers to their practical question: who will be a good reviewer for this paper?

So, they will probably stick to the old practice: try to get a good group of reviewers and ask them to do it. However, this way of working is based on the assumption that being a good reviewer is a long-lasting quality, so that doing a good review predicts that the next review will also be good.

I could not find a clear answer to that question in this paper. I think that with their dataset the authors can probably provide us with an answer that will reassure editors on their decision to stick to the group of reviewers that have produced good reviews in the past.

Would they be so kind?
